# Peroxynitrite formation and sinusoidal endothelial cell injury during acetaminophen-induced hepatotoxicity in mice

**DOI:** 10.1186/1476-5926-2-S1-S46

**Published:** 2004-01-14

**Authors:** Tamara R Knight, Hartmut Jaeschke

**Affiliations:** 1 Department of Pharmacology and Toxicology, University of Arkansas for Medical Sciences, Little Rock, Arkansas 72205, USA; 2 Liver Research Institute, University of Arizona College of Medicine, Room 6309, 1501 N. Campbell Avenue, Arizona 85724, USA

## Abstract

**Introduction:**

Vascular injury and accumulation of red blood cells in the space of Disse (hemorrhage) is a characteristic feature of acetaminophen hepatotoxicity. However, the mechanism of nonparenchymal cell injury is unclear. Therefore, the objective was to investigate if either Kupffer cells or intracellular events in endothelial cells are responsible for the cell damage.

**Results:**

Acetaminophen treatment (300 mg/kg) caused vascular nitrotyrosine staining within 1 h. Vascular injury (hemorrhage) occurred between 2 and 4 h. This paralleled the time course of parenchymal cell injury as shown by the increase in plasma alanine aminotransferase activities. Inactivation of Kupffer cells by gadolinium chloride (10 mg/kg) had no significant effect on vascular nitrotyrosine staining, hemorrhage or parenchymal cell injury. In contrast, treatment with allopurinol (100 mg/kg), which prevented mitochondrial injury in hepatocytes, strongly attenuated vascular nitrotyrosine staining and injury.

**Conclusions:**

Our data do not support the hypothesis that acetaminophen-induced superoxide release leading to vascular peroxynitrite formation and endothelial cell injury is caused by activated Kupffer cells. In contrast, the protective effect of allopurinol treatment suggests that, similar to the mechanism in parenchymal cells, mitochondrial oxidant stress and peroxynitrite formation in sinusoidal endothelial cells may be critical for vascular injury after acetaminophen overdose.

## Introduction

Acetaminophen (AAP) is an effective and safe pain-relieving drug when therapeutic doses are taken. However, an overdose of AAP causes centrilobular necrosis, which in severe cases can lead to liver failure, in both experimental animals and humans [1,2]. It is well established that formation of a reactive metabolite, presumably *N*-acetyl-*p*-benzoquinone imine (NAPQI), by microsomal P450 isoenzymes, is essential for the development of AAP-induced liver toxicity [2]. NAPQI is readily conjugated with glutathione and excreted from hepatocytes [3]. However, excessive NAPQI formation results in covalent binding to sulfhydryl groups of proteins [2,4].

Although protein binding is a critical early event in AAP hepatotoxicity, this mechanism alone cannot explain the severe cell injury. Therefore, several amplifying mechanisms have been postulated. AAP treatment leads to Kupffer cell activation [5] and recruitment of neutrophils into the liver [6]. Furthermore, AAP metabolism causes mitochondrial dysfunction [7-9], which results in mitochondrial oxidant stress [10] and peroxynitrite formation [11]. Recently, we could show that selective scavenging of peroxynitrite with glutathione (GSH) effectively protects parenchymal cells in the liver against AAP-induced cell injury despite continued mitochondrial oxidant stress [12]. This suggests that peroxynitrite plays a critical role in the mechanism of AAP-induced hepatocellular toxicity.

Microvascular disturbances and injury may also be relevant for the progression of AAP-induced liver injury. Walker et al. described sinusoidal endothelial cell (SEC) injury with trapping of red blood cells in the space of Disse (hemorrhage) during AAP-induced liver injury in mice [13,14]. Recently, Ito et al. demonstrated SEC swelling and impaired endothelial scavenger function, which preceded parenchymal cell injury [15]. Subsequent accumulation of erythrocytes in the space of Disse and reduced sinusoidal blood flow indicate substantial microvascular dysfunction [15]. These microvascular changes may cause ischemic injury, platelet aggregation and thrombosis, neutrophil accumulation and inflammatory injury [16]. Severe hemorrhage can cause hypovolemic shock [17]. However, the mechanism and pathophysiological relevance of these events for AAP-induced liver injury remain unclear. Activated Kupffer cells can generate reactive oxygen and nitric oxide [18] and may cause vascular injury [19]. Kupffer cells have been implicated in the mechanism of hepatocellular injury and peroxynitrite formation after AAP overdose [20,21]. Therefore, the objective of this investigation was to test the hypothesis that Kupffer cells may cause SEC injury through formation of vascular peroxynitrite formation.

## Results

**Figure 1:**
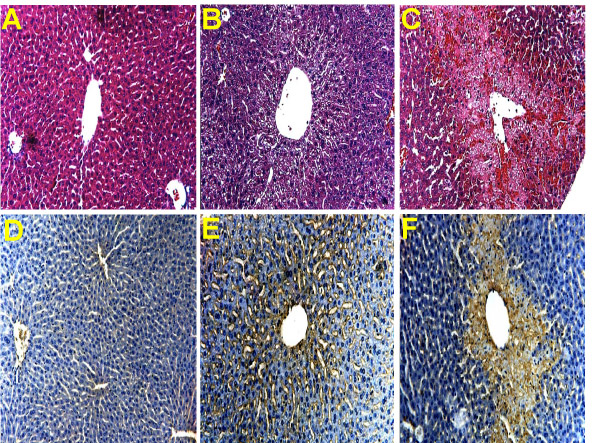
Liver sections were stained with H&E (A-C) or analyzed for nitrotyrosine (NT) protein adducts (D-F). Untreated controls (A,D) are compared to animals treated with 300 mg/kg acetaminophen (AAP) for 1 h (B,E) or 6 h (C,F). Controls**(A) **and 1 h AAP**(B)**: The liver was histologically normal. 6 h AAP**(C)**: Confluent areas of necrosis were seen around all centrilobular regions. Extensive hemorrhage was present. Control**(D)**: No evidence of NT staining. 1 h AAP**(E)**: NT staining localized in vascular lining cells. 6 h AAP**(F)**: Confluent centrilobular NT staining present in hepatocytes.

**Figure 2:**
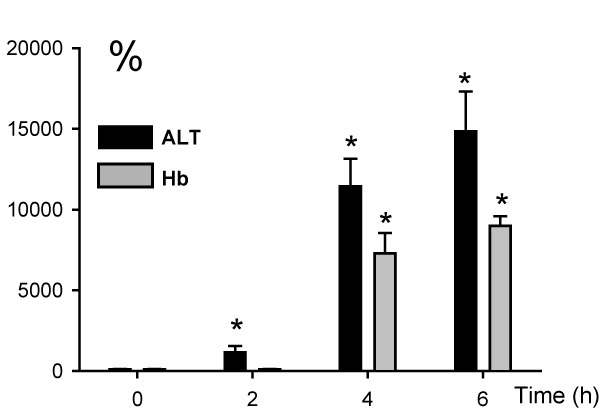
Time course of liver injury (plasma ALT activities) and hemorrhage (liver hemoglobin content) after administration of 300 mg/kg acetaminophen (AAP). Values are given as percent of baseline (ALT: 35 – 8 U/L; Hb: 0.4 – 0.1 mg/g protein). Data represent means – SE of n = 5 animals per time point. *P &lt; 0.05 (compared to untreated control).

**Figure 3:**
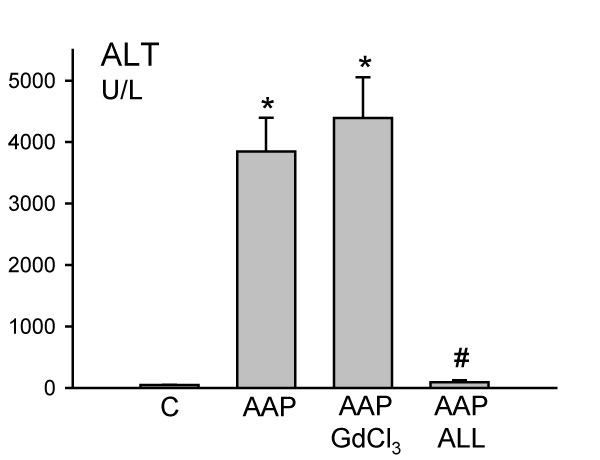
Effect of the macrophage inhibitor GdCl_3 _and allopurinol on acetaminophen (AAP)-induced liver injury (plasma ALT activities). Animals were treated with 300 mg/kg acetaminophen and killed after 6 h. Groups of animals were pretreated with 10 mg/kg GdCl_3 _for 24 h or 2 doses of 100 mg/kg allopurinol (ALL) at 18 h and 1 h before AAP administration. Data represent means – SE of n = 5 animals per time point. *P &lt; 0.05 (compared to untreated control); ^#^P &lt; 0.05 (compared to AAP alone)

**Figure 4:**
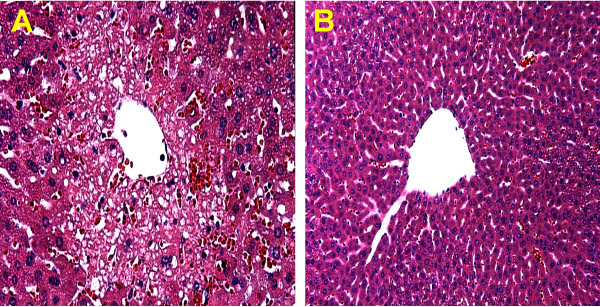
Liver sections were stained with H&E 6 h after administration of 300 mg/kg acetaminophen (AAP). One group of animals was pretreated with 10 mg/kg gadolinium chloride (A), the other group was pretreated with 100 mg/kg allopurinol (B). AAP-GdCl_3_**(A)**: Livers exhibited severe hemorrhage and centrilobular necrosis. AAP-Allopurinol**(B)**: The liver was histologically normal.

**Figure 5:**
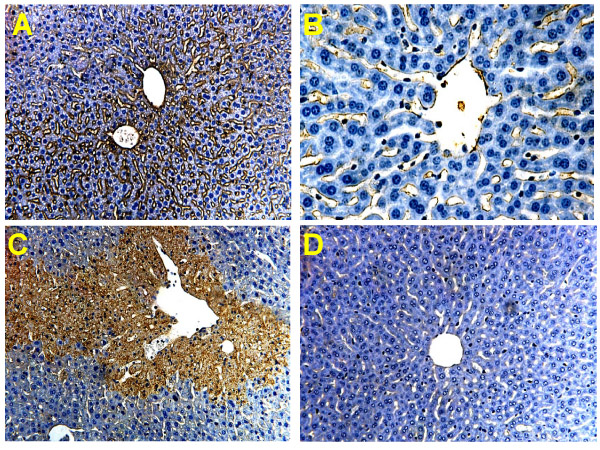
Immunohistochemical analysis of liver sections for nitrotyrosine (NT) protein adducts 1 and 6 h after administration of 300 mg/kg acetaminophen (AAP). One group of animals was pretreated with 10 mg/kg gadolinium chloride (A), the other group was pretreated with 100 mg/kg allopurinol (B). 1 h AAP-GdCl_3_**(A)**: The liver was histologically normal but showed extensive staining in sinusoidal endothelial cells. 1 h AAP-Allopurinol**(B)**: The liver was histologically normal with very limited NT staining in sinusoidal endothelial cells. 6 h AAP-GdCl_3_**(C)**: Liver sections showed centrilobular necrosis and confluent hepatocellular staining for NT in centrilobular areas. 6 h AAP-Allopurinol**(D)**: The liver was histologically normal with no evidence of NT staining.

A dose of 300 mg/kg AAP had no effect on liver tissue at 1 h but caused severe centrilobular necrosis with hemorrhage at 6 h (Figure 1). Red blood cells were trapped in the space of Disse due to sinusoidal cell injury. The large number of red blood cells in the liver was responsible for the increased hepatic hemoglobin content at that time (Figure 2). The time course of hemoglobin accumulation indicated that the sinusoidal cell injury occurred between 2 and 4 h after AAP administration (Figure 2). Thus, the damage to the vascular lining cells developed parallel to the parenchymal cell injury [11,22]. Immunohistochemical staining for nitrotyrosine, an indicator for peroxynitrite formation, demonstrated selective staining of vascular lining cells at 1 h after AAP (Figure 1). However, at 6 h, staining of centrilobular hepatocytes was evident. To test if Kupffer cells may be responsible for the oxidant stress and injury, animals were pretreated with GdCl_3 _to inactivate Kupffer cells. GdCl_3 _treatment had no significant effect on liver injury as indicated by high plasma ALT values (Figure 3). Animals treated with GdCl_3 _still had severe hemorrhage and centrilobular necrosis (Figure 4). On the other hand, pretreatment with allopurinol, which previously was shown to protect against AAP-induced parenchymal cell injury by preventing mitochondrial injury [11], completely eliminated the increase in plasma ALT values (Figure 3) and evidence of necrosis (Figure 4). Furthermore, allopurinol treatment prevented hemorrhage, which suggests that it also prevented sinusoidal endothelial cell injury. Similarly, nitrotyrosine staining as indicator for AAP-induced peroxynitrite formation was neither attenuated in vascular endothelial cells at 1 h nor in parenchymal cells at 6 h by GdCl_3 _treatment (Figure 5). On the other hand, allopurinol treatment prevented endothelial cell and parenchymal cell nitrotyrosine staining (Figure 5).

## Discussion

The objective of this investigation was to test the hypothesis that Kupffer cell-derived reactive oxygen and peroxynitrite could be responsible for vascular and parenchymal cell injury. Previous studies suggested that Kupffer cells are activated after AAP overdose [5] and are relevant contributors to the overall liver injury in rats [20]. More recently, a similar conclusion was reached using the mouse model [21]. However, the reported baseline ALT activities for GdCl_3_-pretreated animals did not correlate with histology, which still showed severe centrilobular necrosis [21]. Our data indicate that Kupffer cells play at most a minor role in the pathophysiology. Gadolinium chloride (GdCl_3_), which functionally inactivates Kupffer cells to produce less reactive oxygen [23], neither reduced the early vascular nor the later parenchymal cell staining for nitrotyrosine. Moreover, GdCl_3 _treatment had no significant effect on vascular cell injury (hemorrhage) or hepatocellular necrosis. These findings are consistent with recent preliminary data showing no attenuation of nitrotyrosine staining or injury in phox-deficient mice, which have no functional NADPH oxidase, the main superoxide producing enzyme in Kupffer cells [24]. In addition to this direct evidence against the involvement of Kupffer cells in the murine model of AAP-induced liver injury, there are other observations that argue against this hypothesis. In general, the most active Kupffer cells are located in the periportal areas [25,26]. Activation of these cells results in a predominantly periportal to midzonal injury [27]. In contrast, AAP causes a strict centrilobular necrosis and hemorrhage (Figure 1) with the earliest and most severe injury affecting the cells closest to the central vein [11,22]. Thus, overall our results are consistent with a number of observations, which do not support a role of Kupffer cells in vascular peroxynitrite formation and injury.

Another potential source of vascular oxidant stress could be infiltrating neutrophils [28,29]. These phagocytes are recruited into the liver in response to the injury [6], i.e., several hours after the occurrence of vascular nitrotyrosine staining [11]. No evidence for a systemic activation of neutrophils was found at any time after AAP treatment [6]. In addition, antibodies against CD18, the common subunit of beta_2 _integrins, which functionally inactivate hepatic neutrophils [30,31], had no effect on AAP-induced hepatotoxicity [6]. These findings suggest that neutrophils are not a relevant source of reactive oxygen species in the vasculature after AAP overdose.

In parenchymal cells, AAP induces mitochondrial swelling [32] and dysfunction [7,8], oxidant stress [10], cytochrome c release [9], peroxynitrite formation [11] and a reduction in cellular ATP levels [10]. Preventing mitochondrial dysfunction with allopurinol treatment eliminated the oxidant stress, peroxynitrite formation and cell injury [10,11]. On the other hand, if peroxynitrite was scavenged by GSH, injury was attenuated despite continued mitochondrial dysfunction [12]. The findings suggest that peroxynitrite is a critical mediator of AAP-induced liver injury. Our present data show that allopurinol treatment prevented the vascular nitrotyrosine staining and hemorrhage. It was previously shown that AAP caused severe depletion of GSH and injury in cultured sinusoidal endothelial cells [33]. These observations document the capacity of sinusoidal endothelial cells to metabolically activate AAP. Since NAPQI, the reactive metabolite of AAP, is responsible for the mitochondrial oxidant stress and peroxynitrite formation in parenchymal cells [11], and the fact that allopurinol prevented vascular nitrotyrosine staining and injury suggests that AAP may have caused a mitochondrial oxidant stress and peroxynitrite formation in endothelial cells. Thus, endothelial cell damage and hemorrhage occurred parallel to the parenchymal cell injury through similar mechanisms.

What is the pathophysiological relevance of the vascular injury in the liver? Without interventions, the massive hemorrhage can lead to hypovolemic shock and death, as shown in other models of sinusoidal endothelial cell injury [17]. However, the early vascular injury between 2 and 4 h had no effect on hepatic ATP levels [10]. These results suggest that the initial hemorrhage does not lead to significant tissue ischemia.

Nevertheless, injury and prolonged dysfunction of sinusoidal endothelial cells, even without severe hemorrhage, can be expected to have a negative impact on liver function. Sinusoidal endothelial cells have not only a barrier function in the liver vasculature but play an important role in clearing a large number of macromolecules and colloids from the circulation. Collagen-, mannose-, Fc gamma-, and hyaluranon scavenger receptors are vital for the turnover of extracellular matrix proteins and the removal of immune complexes [34]. Reduced uptake of formaldehyde-treated serum albumin as early as 2 h after AAP administration demonstrated dysfunction of the hyaluranon scavenger receptor [15].

## Conclusions

Our data argue against Kupffer cells as relevant source of vascular oxidant stress during AAP-induced sinusoidal endothelial cell injury. Our data suggest that, similar to the mechanism in parenchymal cells, mitochondrial oxidant stress and peroxynitrite formation may be critical for sinusoidal endothelial cell injury.

## Methods

### Animals

Male C3Heb/FeJ mice with an average weight of 18 to 20 g were purchased from Jackson Laboratory (Bar Harbor, Maine) and housed in an environmentally controlled room with 12 h light/dark cycle. The animals had free access to food (certified rodent diet no. 8640, Harlan Teklad, Indianapolis, IN) and water. The experimental protocols followed the criteria of the University of Arkansas for Medical Sciences and the National Research Council for the care and use of laboratory animals in research. All animals were fasted overnight before the experiments. Animals received an intraperitoneal injection of 300 mg/kg AAP (Sigma Chemical Co., St. Louis, MO). AAP was dissolved in warm saline (15 mg/ml). Some groups of animals were pretreated with 10 mg/kg gadolinium chloride (GdCl_3_) or 10 ml/kg saline i.v. 24 h before AAP [27]. Other animals received 100 mg/kg allopurinol or 20 ml/kg water p.o. 18 h and 1 h before AAP administration [10,11].

### Experimental Protocols

At selected times after AAP treatment, animals were killed by cervical dislocation. Blood was drawn from the vena cava into heparinized syringes and centrifuged. The plasma was used for determination of alanine aminotransferase (ALT) activities (Test Kit DG 159-UV (Sigma Chem. Co., St. Louis, MO) and expressed as IU/liter. Immediately after collecting the blood, the livers were excised and rinsed in saline. A section from each liver was placed in 10% phosphate buffered formalin to be used in histochemical analyses. A portion of the remaining liver was frozen in liquid nitrogen and stored at -80 degrees C for later hemoglobin determination as described in detail [6].

### Histology and immunohistochemistry

Formalin-fixed tissue samples were embedded in paraffin and 5 micrometer sections were cut. Replicate sections were stained with hematoxylin and eosin (H&E) for evaluation of necrosis [35]. Nitrotyrosine staining was assessed by immunohistochemistry with the DAKO LSAB Peroxidase Kit (K684) (DAKO Corp., Carpinteria, CA) as described [12]. The anti-nitrotyrosine antibody was obtained from Molecular Probes (Eugene, OR).

### Statistics

All results were expressed as mean – SE. Comparisons between multiple groups were performed with one-way ANOVA followed by Bonferroni *t *test. If the data were not normally distributed, we used the Kruskal-Wallis Test (nonparametric ANOVA) followed by Dunn's Multiple Comparisons Test. P &lt; 0.05 was considered significant.
